# Supplementary material for: CRISPR/Cas9-targeted mutagenesis of OsERA1 confers enhanced responses to abscisic acid and drought stress and increased primary root growth under nonstressed conditions in rice
Source: PLoS One. 2020 Dec 3;15(12):e0243376. doi: 10.1371/journal.pone.0243376 (PMC7714338; doi:10.1371/journal.pone.0243376)
Supplement: S1 Fig — Representative photographs of WT plants exposed to mild drought stress for 58 days after sowing. Scale bars = 10 cm. (PDF) [file pone.0243376.s004.pdf]

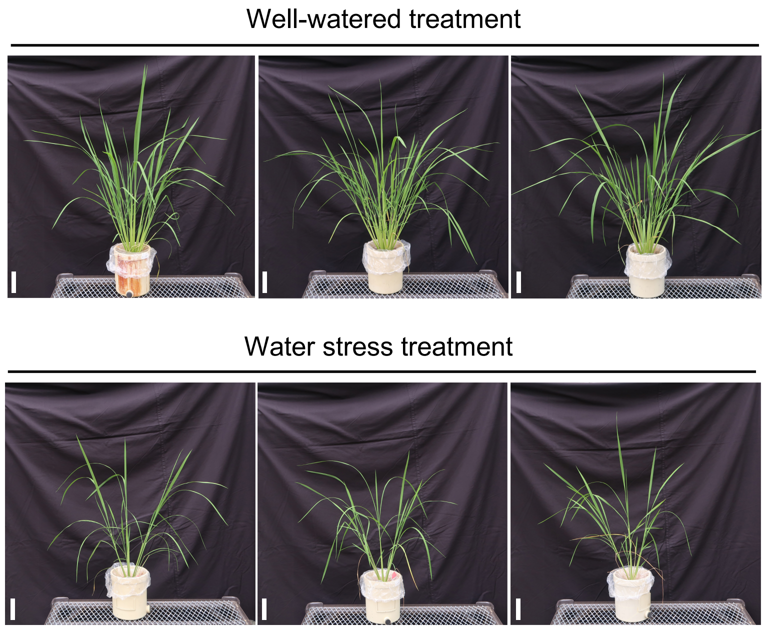

S1 Fig. Growth retardation and reppression of rice plants in the mild drought stress test. Representative photographs of WT plants exposed to mild drought stress for 58 days after sowing. Scale bars = 10 cm.
