# Supplementary material for: CRISPR/Cas9-targeted mutagenesis of OsERA1 confers enhanced responses to abscisic acid and drought stress and increased primary root growth under nonstressed conditions in rice
Source: PLoS One. 2020 Dec 3;15(12):e0243376. doi: 10.1371/journal.pone.0243376 (PMC7714338; doi:10.1371/journal.pone.0243376)
Supplement: S2 Fig — (A) Deletions at the border of the third exon of alleles identified from a sequence analysis of PCR amplicons from the osera1 mutant lines M4 and M5. The deleted bases of the alleles and the protospacer-adjacent motif (PAM) are in red and blue, respectively. (B) Growth phenotype of seedlings of the homozygous and heterozygous progenies of the M4 mutant lines, M4-HM and M4-HT, with deletions at the gRNA2 target site. Seeds were germinated and grown hydroponically for two weeks; representative plants are shown. Scale bar = 1 cm. (C) Growth phenotype of seedlings of the homozygous and heterozygous progenies of the M5 mutant lines, M5-HM and M5-HT, with deletions at the gRNA2 target site. Seeds were germinated and grown hydroponically for two weeks; representative plants are shown. Scale bar = 1 cm. (PDF) [file pone.0243376.s005.pdf]

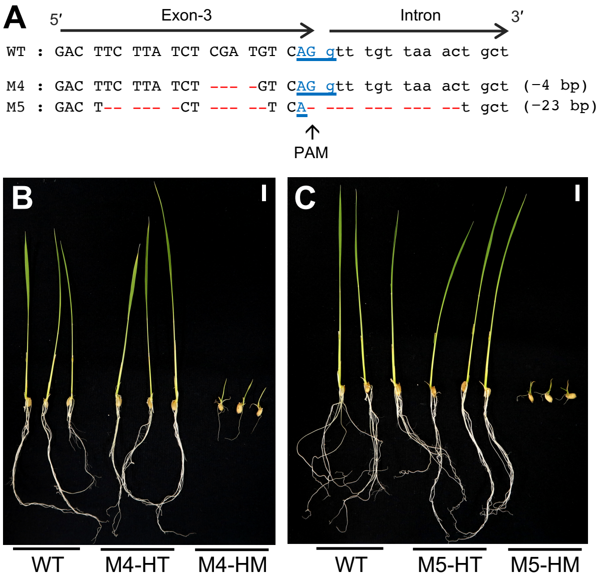

S2 Fig. The growth of homozygous progenies of M4 and M5 mutant lines was arrested at the plumule stage. (A) Deletions at the border of the third exon of alleles identified from a sequence analysis of PCR amplicons from the *osera1* mutant lines M4 and M5. The deleted bases of the alleles and the protospacer-adjacent motif (PAM) are in red and blue, respectively. (B) Growth phenotype of seedlings of the homozygous and heterozygous progenies of the M4 mutant lines, M4-HM and M4-HT, with deletions at the gRNA2 target site. Seeds were germinated and grown hydroponically for two weeks; representative plants are shown. Scale bar = 1 cm. (C) Growth phenotype of seedlings of the homozygous and heterozygous progenies of the M5 mutant lines, M5-HM and M5-HT, with deletions at the gRNA2 target site. Seeds were germinated and grown hydroponically for two weeks; representative plants are shown. Scale bar = 1 cm.
