# Supplementary material for: CRISPR/Cas9-targeted mutagenesis of OsERA1 confers enhanced responses to abscisic acid and drought stress and increased primary root growth under nonstressed conditions in rice
Source: PLoS One. 2020 Dec 3;15(12):e0243376. doi: 10.1371/journal.pone.0243376 (PMC7714338; doi:10.1371/journal.pone.0243376)
Supplement: S3 Fig — Dye exclusion experiments were performed using leaves of 12-day-old seedlings as described by Cui et al. (2019). (A) Before the treatment. (B) Thirty minutes after immersion of the leaves in 0.05% (w/v) toluidine blue solution. No significant difference in cuticle permeability was observed between the leaves of the four osera1 lines, M1T, M1G, M2T, and M3T, and the WT. Scale bars = 1 cm. (PDF) [file pone.0243376.s006.pdf]

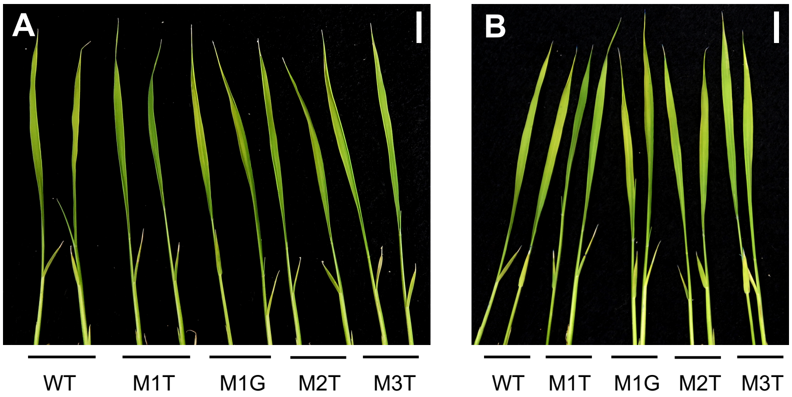

S3 Fig. A cuticle permeability assay for the *osera1* lines. Dye exclusion experiments were performed using leaves of 12-day-old seedlings as described by Cui *et al.* (2019). (A) Before the treatment. (B) Thirty minutes after immersion of the leaves in 0.05% (w/v) toluidine blue solution. No significant difference in cuticle permeability was observed between the leaves of the four *osera1* lines, M1T, M1G, M2T, and M3T, and the WT. Scale bars = 1 cm.
